# ExaCT: automatic extraction of clinical trial characteristics from journal publications

**DOI:** 10.1186/1472-6947-10-56

**Published:** 2010-09-28

**Authors:** Svetlana Kiritchenko, Berry de Bruijn, Simona Carini, Joel Martin, Ida Sim

**Affiliations:** 1 Institute for Information Technology, National Research Council, Ottawa, Ontario, Canada; 2 University of California San Francisco, San Francisco, CA, USA

## Abstract

**Background:**

Clinical trials are one of the most important sources of evidence for guiding evidence-based practice and the design of new trials. However, most of this information is available only in free text - e.g., in journal publications - which is labour intensive to process for systematic reviews, meta-analyses, and other evidence synthesis studies. This paper presents an automatic information extraction system, called ExaCT, that assists users with locating and extracting key trial characteristics (e.g., eligibility criteria, sample size, drug dosage, primary outcomes) from full-text journal articles reporting on randomized controlled trials (RCTs).

**Methods:**

ExaCT consists of two parts: an information extraction (IE) engine that searches the article for text fragments that best describe the trial characteristics, and a web browser-based user interface that allows human reviewers to assess and modify the suggested selections. The IE engine uses a statistical text classifier to locate those sentences that have the highest probability of describing a trial characteristic. Then, the IE engine's second stage applies simple rules to these sentences to extract text fragments containing the target answer. The same approach is used for all 21 trial characteristics selected for this study.

**Results:**

We evaluated ExaCT using 50 previously unseen articles describing RCTs. The text classifier (*first stage*) was able to recover 88% of relevant sentences among its top five candidates (top5 recall) with the topmost candidate being relevant in 80% of cases (top1 precision). Precision and recall of the extraction rules (*second stage*) were 93% and 91%, respectively. Together, the two stages of the extraction engine were able to provide (partially) correct solutions in 992 out of 1050 test tasks (94%), with a majority of these (696) representing fully correct and complete answers.

**Conclusions:**

Our experiments confirmed the applicability and efficacy of ExaCT. Furthermore, they demonstrated that combining a statistical method with 'weak' extraction rules can identify a variety of study characteristics. The system is flexible and can be extended to handle other characteristics and document types (e.g., study protocols).

## Background

Randomized controlled trials (RCTs) are one of the most valuable sources of evidence for the practice of medicine [1]. They are also abundant. Tens of thousands of new RCT findings are published every year. However, most of them are published only as text articles. Because the contents of text articles are not directly computable, this limits the ways in which computers can help analyze and synthesize the voluminous findings from RCTs [2], or the ability of computers to directly reason about these findings in clinical decision support systems [3]. The overall result of this bottleneck is inefficiencies and missed opportunities for using the power of computers to help care providers translate evidence into improved practice.

The need for computable representations of RCTs dovetails with a movement towards open data in science [4] and the reporting of "basic results" in ClinicalTrials.gov [5]. Yet study results are useful only if clear and complete information about the design and execution of the original studies is available, to allow for proper interpretation of potential sources of bias. In the Human Studies Database (HSDB) Project, we are federating the computable description of trial design, execution, and results to support large-scale data analysis and synthesis across many ongoing and completed studies from many different data sources (e.g., publications, institutional registries) for many different purposes (e.g., data mining, new trial design, comparative effectiveness research) [6]. We use the Ontology of Clinical Research (OCRe) as the semantic standard for human studies [7]. OCRe captures trial characteristics such as the parameters of experimental and control interventions, primary and secondary outcomes, population description, funding sources, and publication details, and standardizes these characteristics against standard vocabularies (e.g., SNOMED) and information models (e.g., HL7). OCRe is more detailed and formally structured than the data model used by ClinicalTrials.gov.

There exists the need, therefore, to extract key trial characteristics from full-text journal articles, whether into standardized databases such as HSDB or into local databases or spreadsheets for evidence synthesis projects such as systematic reviews and meta-analyses. Automated methods for this extraction would reduce the time and labor cost compared to current manual methods [8], and would benefit a wide range of users who need to summarize RCT information from full-text journal articles.

In this paper, we present a fully operational system, called ExaCT, that assists human reviewers - who we will henceforth call "curators" - in excerpting sentences and fragments of text describing 21 key trial characteristics (Table 1) - which we will henceforth call "information elements" - from journal publications on RCTs. ExaCT consists of a web browser-based user interface integrated with an automatic information extraction (IE) engine. The IE engine extracts sentences and fragments of text from the journal articles as descriptors of the information elements of interest. The user interface allows the curator to review and modify these excerpts prior to saving the data for coding using OCRe, or for other purposes.

**Table 1:** Target trial characteristics (information elements)

Element	Description
Eligibility criteria	logical conditions for being included in the trial, usually split into inclusion and exclusion criteria

Sample size	the total number of participants actually enrolled (randomized) in the trial

Start date of enrolment	date the enrolment actually started, including day, month, year or as much as presented

End date of enrolment	date the enrolment actually ended, including day, month, year or as much as presented

Name of experimental treatment	name of experimental intervention

Name of control treatment	name of control intervention

Dose	dosage of experimental/control intervention

Frequency of treatment	frequency of administration of experimental/control intervention

Route of treatment	route of administration of experimental/control intervention

Duration of treatment	duration of administration of experimental/control intervention

Primary outcome name	the outcome(s) of greatest importance, where outcome is a "component of a participant's clinical and functional status after an intervention has been applied, that is used to assess the effectiveness of an intervention" (source: Glossary of Terms in the Cochrane Collaboration)

Primary outcome time point	point in time when a primary outcome was assessed

Secondary outcome name	outcome(s) used to evaluate additional effects of the intervention deemed a priori as being less important than the primary outcomes (source: Glossary of Terms in the Cochrane Collaboration)

Secondary outcome time point	point in time when a secondary outcome was assessed

Funding organization name	name of a funding source

Funding number	funding grant number

Early stopping	whether the trial was stopped earlier

Registration identifier of trial	trial registration ID, often ClinicalTrials.gov NCT number

Author name	first and last name of the first author

Date of publication	year the article was published

DOI	digital object identifier for the publication

ExaCT was specifically designed to (1) work on full-text articles and not just abstracts, (2) extract a wide selection of information elements while using one unified approach, and (3) provide an integrated, interactive support to curators. It is through this combination of specifications that ExaCT extends previously reported extraction tools designed for the medical domain [9-16].

In the last decade, a considerable portion of the IE research effort has focused on the biomedical domain (for recent literature reviews see [17] and [18]). Several researchers have investigated the techniques to extract study characteristics and results as well as other important facts from biomedical publications [9-16,19]. Chen et al. used BioMedLEE and MedLEE systems to extract disease-drug associations from biomedical abstracts and discharge summaries [19]. Demner-Fushner and Lin applied classification and extraction techniques to summarize clinical studies reported in journal abstracts in the PICO ("Patient-Intervention-Comparison-Outcome") template [9]. Other researchers applied similar methods for extracting information from meta-sources such as the BMJ compendium of summaries in "Clinical Evidence" [10] and "Cochrane Reviews" [11]. Another source of clinical evidence is RCTs. Much work has been devoted to extracting key trial elements, namely population description, interventions, and outcomes, from RCT publications [12-16]. Overall, the applied extraction techniques rely heavily on manually designed or cue-word-based classification/extraction rules and the use of medical lexicons, such as UMLS, MeSH, and Semantic Groups.

The likelihood of correct information extraction can be improved by narrowing the textual context of the search. One way to narrow the context is by recovering the rhetorical structure of an abstract [20-24] or a full-text article [25,26]. In this approach, each sentence in an abstract/body gets classified as belonging to one of several groups, typically, 'introduction', 'methods', 'results', or 'conclusion'. Although the rhetorical structure of a document does not directly map to a PICO-like structure, it can help locate the PICO elements. It can be observed that most elements will often be found in the 'methods' section and rarely encountered in other sections, which would allow filtering out or down-weighting the irrelevant parts of the document. However, more fine grained classification is necessary to identify the highly specific target parts, such as sentences describing 'patient population', 'interventions', or 'outcomes' [27]. The most-used sentence classification techniques include state-of-the-art statistical learning algorithms, Support Vector Machines (SVM), Hidden Markov Models (HMM), and Conditional Random Fields (CRF), applied on a set of lexical features (words, n-grams, part-of-speech tags) as well as contextual features (position of the sentence in the abstract discourse, features from previous and next sentences). The work by Hara and Matsumoto empirically confirmed that simple extraction rules perform much better if applied in the context of a sentence as opposed to the full abstract [13]. Paek et al. addressed a general task of semantic parsing of sentences and identifying the semantic roles of the words in a predicate [28]. This extra step can potentially boost the IE performance.

There were two main reasons for us to go beyond previous approaches. First, in the context of the HSDB Project, we needed to look at the full text of publications rather than only the abstract or summary. Abstracts tend not to address various trial characteristics, such as complete eligibility criteria, funding sources, secondary outcomes, and whether the trial was stopped early. These details must be identified and extracted in order to support methodologically rigorous data analysis. Methods that work well on succinct types of text such as abstracts, with their neatly delimited context, do not do as well on a publication's full text. Second, each of the previous approaches focused on a small number (1-4) of information elements, so that coverage of all of our 21 information elements would have required us to implement a range of different techniques.

To overcome these limitations, we proposed a unified approach to extract 21 diverse information elements from full-text RCT publications [29]. We make use of a two-step approach to IE. First, a text classifier selects the sentences in the text that are most likely to contain a particular piece of target information. Then, simple regular expression rules are applied to extract the exact text excerpts from these selected sentences. This strategy was based on the intuition that if the context is sufficiently restricted (e.g. 'this sentence is the most likely one to mention the start date of a trial'), then a simple rule (e.g., 'the first occurrence of a date') is enough to extract the sought-after information. The proposed approach does not require extensive individual modelling for each information element as do methods with a strong semantic and/or linguistic reliance [30]. Our preliminary results showed good performance demonstrating that our statistical classifier for sentence selection, combined with simple ('weak') extraction rules, can address the diversity in the task. Independent of our earlier study, Patwardhan and Riloff [31] exploited a strikingly similar two-stage IE strategy that they found to be beneficial in their application domains (public safety), though their scope and design details differ from ours.

The present work builds on [29] and presents a complete IE system for RCTs, named ExaCT. We extended our previous work in three main directions: system improvement, user interface, and system evaluation. We have refined and extended the core algorithms and pattern rules for pre-processing, sentence classification, fragment extraction, and post-processing. We have designed and implemented a web browser-based curation user interface. Finally, we have performed an evaluation of the entire system.

## Methods

**Figure 1:**
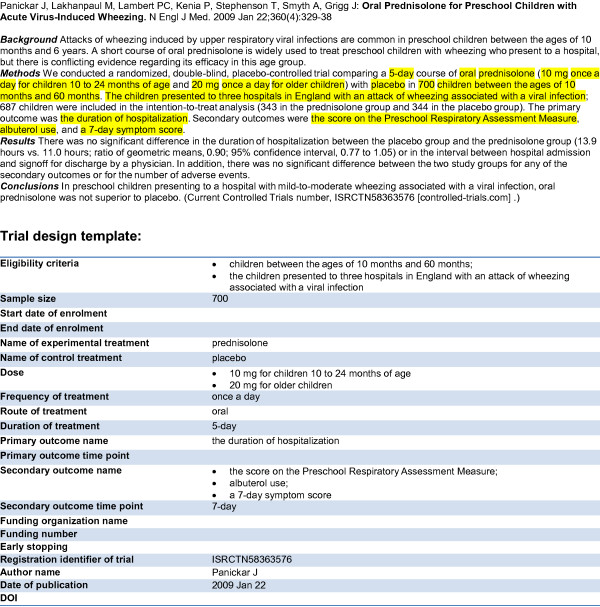
**Example of an abstract and the corresponding template filling**. The top part of the figure shows the abstract of a journal article regarding an RCT. The bottom part shows the template with the slots filled in with text excerpts from the abstract. Some slots are left empty as the information is not present in the abstract.

The key part of the ExaCT system is its IE engine, which extracts pieces of information (sentences and/or text fragments) from a trial publication to fill slots in the pre-defined template. The template includes 21 information elements (Table 1) based on the CONSORT statement [32,33] and on a task analysis of the information needs of systematic reviewing [34]. Fig. 1 presents an example of the template filled in with the information contained in a trial publication abstract. Noticeably, the elements vary greatly in their structure. Some are short, precise pieces of information, e.g. the number of subjects enrolled (*sample size*). Others, such as *eligibility criteria*, are lengthy, free-text descriptions spanning several sentences. Even though all this information is essential for a comprehensive description of a trial, often some parts are skipped (e.g. *start date *and *end date of enrolment*) or poorly defined in a publication (e.g. "main outcomes" instead of a distinction between *primary *and *secondary outcomes*).

**Figure 2:**
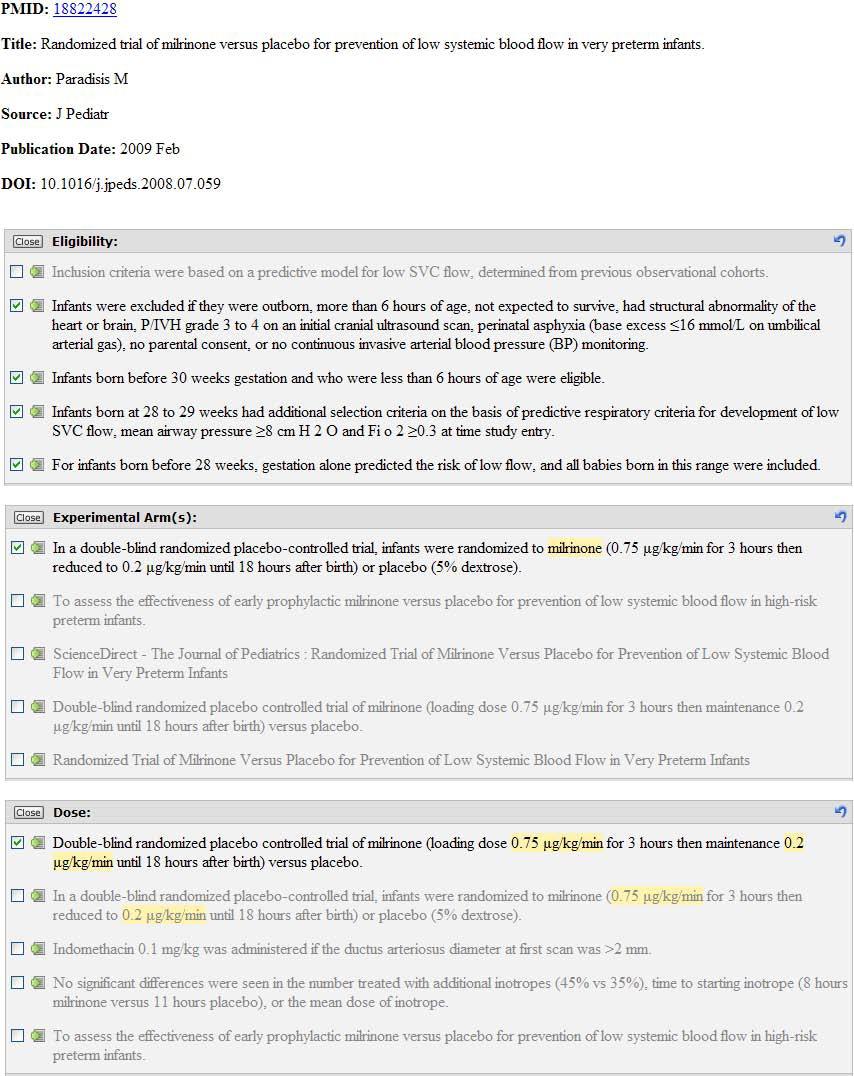
**Example of the system's output**. The publication details of an article are retrieved directly from PubMed. For other information elements, the system outputs five best candidate sentences in decreasing order of confidence. The text fragments identified by the system as containing the target information are highlighted in the retrieved sentences whose confidence score is above a certain threshold. For *eligibility criteria*, the whole sentence is considered the target, so no fragments are highlighted in those sentences. Sentences in black were confirmed as correct answers by the field expert.

ExaCT's IE engine looks for text excerpts that most closely describe the trial information elements of interest. For each element (with the exception of publication details, i.e. *author name*, *date of publication*, and *DOI*, which are retrieved directly from PubMed), the system outputs the five best candidate sentences in decreasing order of confidence (Fig. 2). The text fragments identified as containing the target information on the element are highlighted in the retrieved sentences. If the confidence level of a particular sentence is too low, no text fragments are highlighted, even if the sentence is among the five best. For *eligibility criteria*, the whole sentence is considered the target, so no fragments are highlighted in those sentences. Note that in a publication, for each information element there can be

• no answer, or

• exactly one answer provided by one instance of text, or

• one answer repeated in several instances of text, or

• several distinct answers.

For the current study, all distinct answers have to be identified by the system (e.g. for *eligibility criteria*) while only one answer is required for a set of redundant instances (e.g. for *name of experimental treatment*).

### System design

#### Overall architecture

Our unified approach is based on a machine learning paradigm. Manually labeled training material is collected so that the system can automatically learn the correct context for each information element. Then, a set of hand-crafted 'weak' rules is applied to the identified contexts to extract the exact values for each element. For example, in a sentence that contains enough language clues (i.e. words and phrases) for the system to recognize the context for *start date of enrolment*, the first appearance of a date is returned as the target for this element. This approach relies on two main assumptions. First, a 'weak' extraction rule, too unspecific to extract a precise piece of information from the whole article, will likely be accurate in a narrow enough context (e.g., a sentence). Second, segmentation at the sentence level provides a context that is narrow enough to directly get to the target information and broad enough to correctly judge its relevance.

Fitting this two-step procedure into a general workflow resulted in the following system design:

1. Text pre-processing, including sentence splitting, automatic annotation of common entities, section heading identification, irrelevant section removal;

2. For each information element:

a. Sentence classification/ranking (classification component)

b. Application of 'weak' extraction rules (extraction component)

3. Post-processing of results.

#### Pre-processing

Clinical trial publications come in a range of document standards and formatting schemas, from detailed XML to various forms of HTML, PDF, word processor documents, and even OCR-ed documents in ASCII. PDF or word processor documents are converted into HTML (if possible) or plain text format. The HTML/XML format is preferable as it better preserves the original document structure.

Further pre-processing of a textual document is fully automatic. First, the main sections and subsections of an article are identified. XML documents often have sections and their headings clearly marked with the corresponding tags (e.g. <sec> <title>*section heading *</title>*section content *</sec>). In HTML documents, tags marking section headings are used erratically from journal to journal, but quite consistently within the same article. Assuming this consistency, we employ the following algorithm for section detection. For each article, sequences of HTML tags surrounding the phrases commonly found to be section headings in scientific publications (such as 'Abstract', 'Methods', 'Results') are collected; subsequently, all phrases surrounded by identical or similar tag sequences within the article are assumed to be section headings or subsection headings. As previously noted in [35], the detection of section boundaries is not a trivial task. The above algorithm gives only approximate boundaries for major sections. However, incidental observations indicated that this pre-processing step was often helpful and never harmful. Since this step is a non-critical component of the overall system, we find that an independent evaluation of this algorithm is beyond the scope of the current work.

Next, sections of the article that are irrelevant to the trial description (e.g. references, related articles, editors' notes) are removed. The remaining text is split into sentences, and each sentence is annotated with the section and the nested subsections of origin (e.g. section "Methods" → subsection "Patients"). In addition, several entities are annotated with tags to allow for generalization. The entities include numbers, units, measurements, dates, and people. For example, "17 women participated" gets tagged as " <integer> 17 </integer> <person> women </person> participated".

#### Sentence classification

Sentence classification is built around a statistical machine learning component, based on the Support Vector Machine (SVM) algorithm, which learns a statistical model from articles that a field expert manually annotated. A separate statistical model is created for each information element. Then, at the classification step, each element's model is applied to all sentences to discover which sentences are most similar to the training examples for this element. Within the classification step, a sentence is represented with a bag-of-terms, where terms are words and annotation tags, as well as multi-word phrases (word n-grams). For each information element, the output of the classification stage is a ranked list of the top five sentences scored by a classifier as the most promising to contain the target information. If the confidence score of the top candidate sentence is very low, a "not found" message is shown to the user.

**Figure 3:**
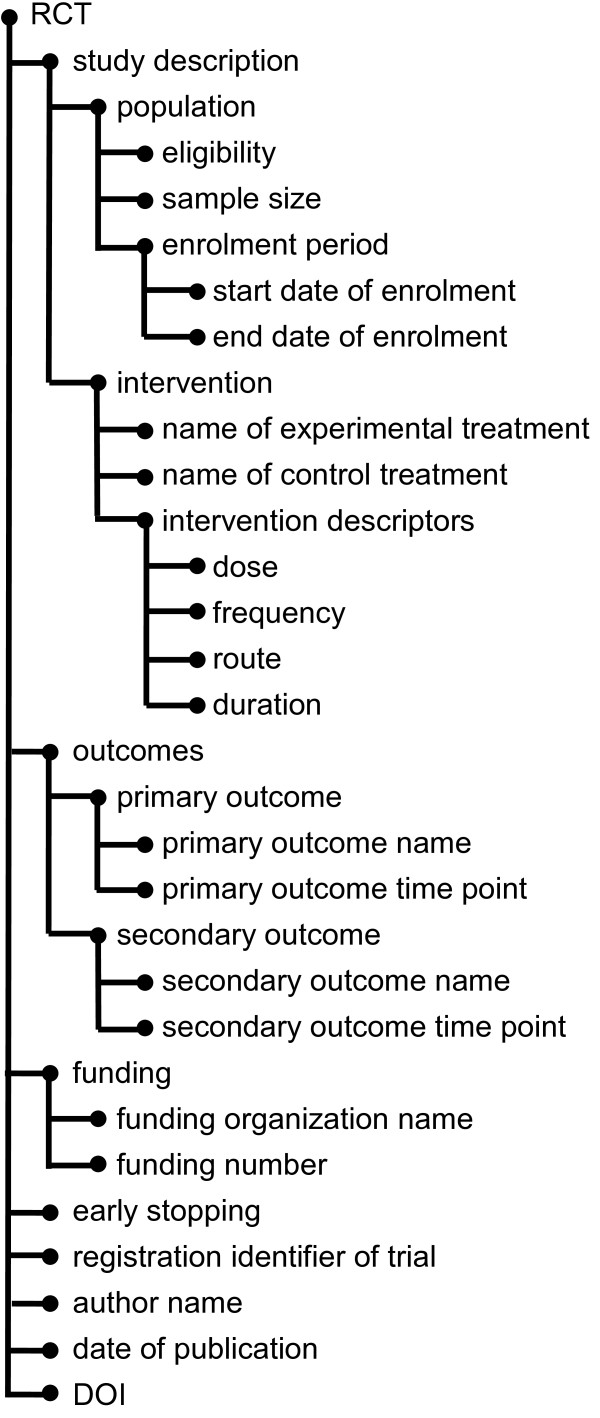
**Hierarchy of information elements**. The hierarchy of the 21 information elements used in ExaCT. The elements are grouped semantically in the four-level hierarchical structure to enhance the sentence classification method.

This conventional classification framework has been enhanced with a hierarchy of elements. The information elements were organized into several groups, namely intervention parameters, population description, outcomes, and funding sources. As the elements in a group are closely related semantically, they also tend to appear together in a sentence. Furthermore, these semantic relations can be propagated to a multi-level hierarchy. The four-level hierarchical structure designed for this project is shown in Fig.3. To take advantage of this hierarchical organization of information elements, we fit our learning component into the probabilistic hierarchical top-down framework [36]. In this framework, a statistical model is learned for each information element (leaf nodes) as well as for each internal node of the hierarchy. Then, the classifiers' confidence scores at all nodes on the path to the element node (the node and its ancestors) are combined to make the final prediction for the information element. In this way, evidence from all related elements is collected to make a better informed decision.

#### Fragment extraction

A set of regular-expression 'weak' extraction rules was manually crafted for each information element. Most of the rules make use of the element's structure, whether it is a number, a date, or a measurement. For example, *start date of enrolment *is extracted as the first string that looks like 'a date', *sample size *is an integer number with a reference to people (patients, women, subjects, etc.), *funding organization *is a sequence of words with the first letter capitalized. Other rules concentrate on a specific context of the information element, e.g. "randomly assigned to receive either *name of experimental treatment *or *name of control treatment*".

Occasionally, none of the common patterns implemented as the 'weak' extraction rules appears in a sentence. In this case, we apply a second procedure that attempts to extract text fragments by looking for redundant information among the top five sentences. Typically in a journal publication, information on key trial characteristics is repeated throughout the article (e.g. in the abstract, the methods section, and the conclusion). The extraction module looks for words and phrases common among the highly scored sentences, filters out known irrelevant phrases, and outputs the rest as the target information. This procedure is appropriate only for elements that tend to be expressed in words and phrases that have no strong underlying structure or pattern, i.e. *primary and secondary outcome names *and *name of experimental treatment*.

In the case of *eligibility criteria*, the whole sentence tends to be the target, so no further fragment extraction needs to be performed.

#### Post-processing

The classification module outputs five top-scored sentences in decreasing order of their confidence scores. A post-processing step was added that boosts the confidence score for sentences with a 'weak' pattern match, as well as those with fragments extracted by the redundancy algorithm. This may result in a re- ordering of the sentences in the top-five list. For example, a sentence *s_1 _*with no date information is less likely to describe the trial's *start date of enrollment *than a slightly lower scoring sentence *s_2 _*with a 'date' string, even if sentence *s_1 _*contains several words and phrases common for this element's context (e.g. "design setting", "were recruited", "trial conducted").

Publication details are readily available in Medline. A separate module within the program links the article to its Medline citation by searching PubMed on title words. It then fetches the structured Medline record and parses from it the relevant information elements.

### User interface (UI)

In our practical context, a critical requirement for an automatic IE system is a user interface that allows a curator to review and, if necessary, to amend the extracted information before further use. Since the system is not perfectly accurate, the assessment and revision step is necessary to ensure the correctness and completeness of the extracted data.

In ExaCT's interface, the information elements are divided into five semantic groups and displayed in separate tabs: publication information (first author, DOI, publication date), meta information (funding sources and trial registration), enrolment (eligibility criteria, sample size, start date and end date of enrolment, whether the trial was stopped early), interventions (including dose, frequency, route, and duration), and outcomes (primary and secondary outcomes and their time points). For each element, only the top-scored solution with a high confidence score or a "not found" message is displayed at first to avoid overwhelming the curator. Later, the curator can view the list of all five system's suggestions and choose one or more sentences as the most relevant to the information element. If a target sentence is not among the system's five candidates, the curator can add sentences from the article. Automatically extracted fragments are highlighted in the sentences. The highlighting can be modified by the curator using the mouse.

**Figure 4:**
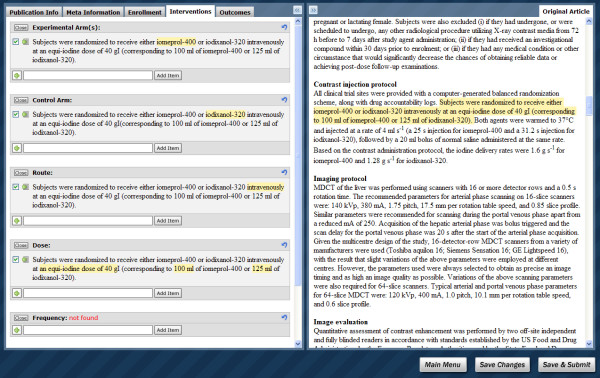
**Interactive user interface for curation**. The user interface consists of two panels simultaneously displayed on a screen: the left panel displays the system's suggestions and the right panel displays the original article. A button next to each sentence on the left panel highlights the same sentence within the article on the right panel. The information elements are divided into five tabs: publication information, meta information, enrolment, interventions, and outcomes. For each element, the top-scored solution with a high confidence score or a "not found" message is initially displayed. A user can expand the list of the system's suggestions to the five highest scoring solutions and choose one or more sentences as the most relevant to the element. If a target sentence is not among the system's five choices, a curator can add a sentence directly from the article by copying-and-pasting or dragging-and-dropping it into the corresponding element text box, or by right-clicking and selecting the relevant element from the menu. Automatically extracted fragments are highlighted in the sentences. The highlighting can be modified by a curator using the mouse.

This basic user interface was a starting point in our evaluation of the IE system. During the evaluation, several modifications were suggested by the users and were implemented in the final version of the UI (Fig. 4). The UI is fully integrated with ExaCT; the program communication is carried out by means of a database. Two panels are simultaneously displayed on the screen: the left panel shows the system's suggestions and the right panel shows the original article. Such a design aims at saving curation time as a curator repeatedly needs to refer to the text of the article. Each suggested sentence can be viewed in a larger context by pressing a button next to it, which highlights the sentence in the original article (see Fig. 4). Viewing a sentence in context helps the curator quickly assess the validity and completeness of the extracted information. A curator can add a sentence directly from the article by (1) copying-and-pasting or (2) dragging-and-dropping it into the corresponding element text area or (3) by right-clicking the selected text and choosing from a menu the element to which the sentence is relevant. When finished reviewing the extracted information, the curator is shown a summary with the selected sentences (for eligibility criteria) and the highlighted fragments (for the rest of the elements) for the final approval before the data are saved in the database.

A video demonstrating the interface features can be found in the additional material section (additional file 1: ExaCTDemo.mp4). The demo version of the system is publicly available at http://exactdemo.iit.nrc.ca.

### Evaluation

#### Data collection

For evaluation purposes, we collected two non-overlapping sets of full-text journal articles that describe RCTs. The first set was used for training the sentence classification component and for devising the hand-crafted extraction rules for the extraction component. Initially, this set contained 78 articles randomly chosen from five core clinical journals: Annals of Internal Medicine, the New England Journal of Medicine, PLoS Clinical Trials, JAMA, and The Lancet. These journals were chosen to be representative of general medicine (i.e., not restricted to a single clinical domain), and because their articles are reasonably complete in describing trial details. The articles were manually annotated by a field expert to delimit the information elements within the text. Later, 54 more articles from a wider pool of journals were added to the training set in a semi-supervised manner: first, the articles were processed by ExaCT and then the system's output was revised by the field expert. Therefore, the total number of articles in the training set was 132 (from 22 clinical journals). The articles from PLoS Clinical Trials were in XML format conforming to the PubMed DTD while all other articles came in journal-specific HTML format.

The second set of articles was used exclusively for testing purposes. That set consisted of 50 full-text articles describing RCTs from 25 medical journals. The articles were selected by one of the authors (SC) using the PubMed search interface. The selection was based on the following criteria. All articles

• were written in English;

• were published in the core clinical journals (as defined by PubMed) in 2009;

• had abstracts and full texts available in HTML format;

• reported on RCTs on human subjects.

Only trials on drug treatments were considered in order to minimize the variability of natural language used to describe the experimental and control conditions. Crossover and cluster-randomized trials were excluded as well as studies that represented phases or sub-studies of trials (e.g., secondary analyses of RCTs). Articles in which the target trial information was presented in tables stored in separate files were also excluded from the set.

The original articles from the test set were processed by ExaCT and the results were presented to a curator (SC) through the user interface. Thirty-eight articles were reviewed with the basic interface and the remaining 12 articles were evaluated on the final version of the UI. The curator assessed and corrected the system's output and her answers were compared with the original solutions. This setup aimed to evaluate the adequacy and usefulness of the system's suggestions for a human curator.

#### System performance evaluation

There are two levels of system performance. The *sentence level *performance concerns the ability of the system to identify sentences carrying relevant information on a particular element. Relevant sentence selection alone significantly aids a curator by drastically narrowing the textual region to consider for target slot filling. The *fragment level *performance represents the ability of the system to correctly fill in the information slots with the sentence fragments. ExaCT's two-level architecture was designed to reflect this two-level performance presentation. The sentence level performance is primarily determined by the sentence classification component. The classification component ranks sentences according to their relevance to a particular information element and outputs the top five candidates. However, the order of those five sentences can be re-arranged by the extraction component, which boosts the relevance scores of the candidates that matched the extraction rules. Finally, the top-scored sentence is presented to a curator as the *system's suggestion *if its score is above a certain threshold. Otherwise, the *system's suggestion *is empty and a "not found" message is shown.

We report different levels of performance with precision (positive predictive value) and recall (sensitivity) measures. The precision is defined as the proportion of returned instances that are truly relevant; and recall is the proportion of relevant instances returned by the system [37]:

$$P_{i}=\frac{TP_{i}}{returned\;instances_{i}};\;R_{i}=\frac{TP_{i}}{relevant\;instances_{i}},$$

where *TP_i _*(true positives) is the number of returned instances that are truly relevant for element *i*. These measures are averaged across *n = 21 *information elements by micro-averaging and macro-averaging:

$$micro-averaged\;P=\frac{\sum_{i=1}^{n}TP_{i}}{\sum_{i=1}^{n}returned\;instances_{i}};$$

$$macro-averaged\;P=\frac{\sum_{i=1}^{n}P_{i}}{n}$$

The formulas for calculating micro- and macro-averages of recall are analogous.

## Results

Table 2 summarizes the sentence level performance. Given that the information value for most elements is contained in a single sentence, we first evaluate the capability of ExaCT to recover at least one relevant sentence for each information element described in a paper (left part of the table). The total number of articles where a trial element is mentioned varies. Some elements, such as *eligibility criteria *and *names of experimental and control treatments*, were always present, while others, such as *registration identifier of trial*, were sometimes absent. Two elements, *funding number *and *early stopping*, were present in only a few articles. Precision and recall for the *system's suggestion *reflect the quality of sentence ranking as well as the system's potential to confirm the absence of element description. The *system's suggestion *was considered truly relevant if it represented a sentence confirmed by the curator to be a correct answer. Averaged over 21 information elements, both precision and recall were 80%. The quality of all top five candidate sentences is evaluated with top5 recall. On average, the top five candidates contain at least one relevant sentence for 93% of the cases. The rightmost section of the table displays the capacity of the system to recover all relevant sentences for each information element, since sometimes more than one sentence is required to convey all essential details on a particular information element. In total, the classification module was able to recover 88% of the 970 relevant sentences among its top five sentence candidates. These results support our assumption that five candidates is a good balance between presenting a manageable amount of information to a curator and recovering sufficient relevant information.

**Table 2:** Sentence level performance of ExaCT

Information element	at least one relevant answer per article				all answers	
	
	# of articles with expert's answers	*system's suggestion*		top5 recall	# of sentences with expert's answers	top5 sentence recall
					
		precision	recall			
Eligibility criteria	50	0.78	0.78	0.98	133	0.77

Sample size	50	0.77	0.68	0.84	52	0.83

Start date of enrolment	37	0.97	0.86	0.86	37	0.86

End date of enrolment	37	0.91	0.81	0.89	37	0.89

Name of experimental treatment	50	0.86	0.86	0.98	56	0.95

Name of control treatment	50	0.86	0.86	1.00	54	0.96

Dose	49	0.81	0.78	0.98	72	0.90

Frequency of treatment	41	0.80	0.78	1.00	55	0.95

Route of treatment	37	0.86	0.81	0.95	39	0.95

Duration of treatment	41	0.74	0.76	0.93	44	0.91

Primary outcome name	48	0.66	0.69	0.88	52	0.81

Primary outcome time point	31	0.53	0.61	0.81	33	0.76

Secondary outcome name	43	0.69	0.79	0.98	49	0.88

Secondary outcome time point	26	0.69	0.69	0.88	29	0.83

Funding organization name	40	0.47	0.50	0.72	42	0.74

Funding number	5	0.31	0.80	0.80	5	0.80

Early stopping	2	0.33	1.00	1.00	2	1.00

Registration identifier of trial	31	1.00	0.94	0.94	31	0.94

Author name	50	0.98	0.98	0.98	50	0.98

Date of publication	50	0.98	0.98	0.98	50	0.98

DOI	48	1.00	0.98	0.98	48	0.98

**Micro-average**	816	**0.80**	**0.80**	**0.93**	970	**0.88**

**Macro-average**		**0.76**	**0.81**	**0.92**		**0.89**

Three data elements were identified with less than 80% recall: *funding organization name*, *eligibility criteria *and *primary outcome time points*; possible reasons for this are reviewed in the Discussion section.

The fragment level performance was evaluated on sentences from the top five candidates selected by a curator as the most relevant for an information element. Table 3 summarizes the extraction results. On average, the trial information slots were filled in with 93% precision and 91% recall. For information elements that tend to be described in complex patterns, e.g. *names of experimental and control treatments *and *primary and secondary outcomes*, the exact fragment boundary identification poses additional challenges to the system. Weak extraction rules designed for those elements often spot the right place in a sentence, but go beyond the correct boundaries including irrelevant information or fall short of including all of the essential details. With partial matches taken into account, precision and recall rise to 96% and 94%, respectively.

**Table 3:** Fragment level performance of ExaCT

Information element	# of expert's fragments	exact match		partial match	
		
		precision	recall	precision	recall
Eligibility criteria	103	1.00	1.00	1.00	1.00

Sample size	46	0.89	0.87	0.89	0.87

Start date of enrolment	32	1.00	1.00	1.00	1.00

End date of enrolment	31	1.00	1.00	1.00	1.00

Name of experimental treatment	54	0.72	0.54	0.97	0.72

Name of control treatment	55	0.83	0.80	0.89	0.85

Dose	103	0.91	0.90	0.96	0.97

Frequency of treatment	70	0.91	0.87	0.99	0.93

Route of treatment	53	0.94	0.92	0.94	0.92

Duration of treatment	45	0.84	0.91	0.86	0.93

Primary outcome name	38	0.97	0.97	0.97	0.97

Primary outcome time point	33	0.90	0.79	0.97	0.85

Secondary outcome name	43	0.93	0.88	1.00	1.00

Secondary outcome time point	25	0.72	0.72	0.92	0.92

Funding organization name	45	0.90	0.98	0.90	0.98

Funding number	7	1.00	1.00	1.00	1.00

Early stopping	2	1.00	1.00	1.00	1.00

Registration identifier of trial	29	1.00	1.00	1.00	1.00

Author name	49	1.00	1.00	1.00	1.00

Date of publication	49	1.00	1.00	1.00	1.00

DOI	47	1.00	1.00	1.00	1.00

**Micro-average**	959	**0.93**	**0.91**	**0.96**	**0.94**

**Macro-average**		**0.93**	**0.91**	**0.96**	**0.95**

The overall performance of ExaCT is presented in Table 4. In two thirds of 1050 IE tasks (21 information element slots for 50 articles), the system provided a fully correct solution, i.e., it identified a sentence with the target information as its top-scored solution and extracted the correct textual fragments to fill in the element slots, or it reported the absence of the solution if an information element was indeed not mentioned in the publication. Among the other one third of solutions, there were partially correct (28%) and incorrect (less than 6%) solutions. The partially correct solutions were those where the correct solution was present among the five choices, but not (only) in the top-scored sentence, and/or the fragment selection in the sentence(s) was incorrect. The incorrect solutions were those where none of the five sentences suggested by the system contained the relevant information on the element.

**Table 4:** Performance of the entire IE system

Information element	fully correct solution	partially correct solution				incorrect solution
			
		total	sentence selection only	change in highlighting	sentence adding	
Eligibility criteria	0.08	0.90	0.50	0.00	0.40	0.02

Sample size	0.56	0.28	0.04	0.24	0.02	0.16

Start date of enrolment	0.88	0.02	0.02	0.00	0.00	0.10

End date of enrolment	0.82	0.10	0.06	0.04	0.00	0.08

Name of experimental treatment	0.38	0.60	0.04	0.56	0.02	0.02

Name of control treatment	0.62	0.38	0.08	0.30	0.02	0.00

Dose	0.50	0.48	0.10	0.32	0.10	0.02

Frequency of treatment	0.60	0.40	0.02	0.34	0.06	0.00

Route of treatment	0.74	0.22	0.04	0.18	0.00	0.04

Duration of treatment	0.58	0.36	0.10	0.26	0.02	0.06

Primary outcome name	0.58	0.30	0.12	0.10	0.08	0.12

Primary outcome time point	0.42	0.46	0.22	0.22	0.02	0.12

Secondary outcome name	0.60	0.38	0.20	0.14	0.06	0.02

Secondary outcome time point	0.56	0.38	0.12	0.24	0.04	0.06

Funding organization name	0.38	0.40	0.26	0.14	0.00	0.22

Funding number	0.80	0.18	0.18	0.00	0.00	0.02

Early stopping	0.92	0.08	0.08	0.00	0.00	0.00

Registration identifier of trial	0.96	0.00	0.00	0.00	0.00	0.04

Author name	0.98	0.00	0.00	0.00	0.00	0.02

Date of publication	0.98	0.00	0.00	0.00	0.00	0.02

DOI	0.98	0.00	0.00	0.00	0.00	0.02

**Micro-average = Macro-average**	**0.66**	**0.28**	**0.10**	**0.15**	**0.04**	**0.06**

The numbers represent the proportion of the 50 test articles for which a fully correct, a partially correct, or no correct solution has been found by the IE engine.

For the curator who was correcting ExaCT's extractions, relatively minor modifications, i.e., to mark sentences as relevant/irrelevant among the top five candidates, were required in 10% of the tasks. Therefore, in 76% of the tasks, the assessment and correction of the automatic results took minimal curation time. The more time consuming operation, requiring the curator to add sentences not found by the system, was necessary in only 4% of the extraction tasks. Mainly, this operation was used for *eligibility criteria *(40% of the articles) and very rarely for other elements.

We measured the time required for a curator to review and correct ExaCT's solutions. With the basic version of the user interface the curation time averaged 9 min 31 sec per article. Using the improved version of the UI, the curator spent on average 7 min 21 sec per article (23% reduction in time). Previously, manual curation took from 8 to 20 hours per article, but it involved extraction and data entry of a considerably larger set of information elements and required fine-grained partitioning of the information to match the structure of the database [38]. Thus, the direct comparison of curation time would be inappropriate, but is useful here as a very preliminary indication of potential savings in time. A large-scale usability study would be required to verify actual time savings.

## Discussion

ExaCT demonstrated very good performance on a test set of 1050 tasks found in 50 articles from a wide range of clinical trial publications (25 journals). The system was able to identify most of the sentences describing the selected information elements and extract the target text fragments from those sentences. A leave-one-out cross-validation on the training set (the results are not reported here) showed similar performance, confirming that our evaluation gives a fair indication of the system's performance in real-life settings. While the system's accuracy is not perfect, it is high enough to provide valuable help and to potentially save a considerable amount of time for a curator.

The evaluation results confirmed the validity of the two assumptions essential for the two-level architecture of ExaCT. The first assumption, that weak extraction rules can identify target pieces of information if the context is reasonably restricted (i.e., in a sentence), held true for all trial elements in the majority of situations. The second assumption, that a sentence provides broad enough context for the system to judge its relevance to a particular information element, generally held true for all but one element we tested. Only *eligibility criteria *tend to be described in a text segment that spans multiple sentences. Nevertheless, for most articles the system was able to recover a large portion of sentences on eligibility criteria individually, one-by-one, as part of the set of five top-scoring sentences.

For one information element, *funding organization names*, a low recall was due to an idiosyncrasy in the test set. In several articles of the test set the sentence describing the funding sources was placed after the reference section. Yet, the training set contained no such articles. The tail of the text (from the reference section downward) had been ignored by the classification component as this part was never found to contain relevant information, while it often did contain misleading extraneous text such as comments and information on related articles and their abstracts. A slight revision of the heuristic, i.e. allowing the classifier to search for a funding source in all parts of an article, would have recovered eight of previously missed sentences and would have increased the recall for *funding organization name *to 90% and for *funding number *to 100%.

The lack of a public benchmark dataset for the task prevents the comprehensive comparison of the results with the previous work. Several researchers attempted to extract trial characteristics, namely population description, study interventions, and outcomes, from journal article abstracts. The reported results range from 52% to 84% for sentence classification [12,13,27] and from 68% to 95% for information extraction [9-11,13-16]. Due to significant differences in the task (e.g., abstracts vs. full texts) and evaluation settings (e.g., exact vs. partial match), these results are not directly comparable to ours. When such a comparison becomes possible, the generic technique used in ExaCT will likely not be as accurate as some specialized approaches that target only a few information elements. If that is true, ExaCT would in effect exchange some accuracy for a uniform solution.

The system evaluation revealed the following weaknesses of the proposed framework.

### Sentence level

#### 1. Classification of sentences whose relevance can be recognized only by their outer context

Limiting the working context to a single sentence can occasionally be overly restrictive. Some sentences provide details on previously mentioned information, but by themselves do not contain any language clues for the classification module to recognize their relevance to a given information element.

Example (*eligibility criteria*):

The presence of atherosclerosis was determined by ≥50% stenosis in at least one coronary artery at cardiac catheterization, by history of previous myocardial infarction, previous angioplasty, previous coronary artery bypass graft surgery, previous ischemic stroke, or documented peripheral arterial disease.

This sentence's association with *eligibility criteria *can be determined only by its proximity to the key eligibility sentence "The study included 30 men with stable atherosclerosis and fasting low-density lipoprotein (LDL) cholesterol levels ≥100 mg/dL off statin therapy."

#### 2. Study outcomes and their respective time points not clearly defined and/or separated into primary and secondary

Several articles did not make a clear distinction between primary and secondary outcomes. Moreover, in a few cases only measurements and assessments performed to evaluate the effects of the studied treatments were noted, without explicit denotation of these measures as outcomes. Since those measurements differ from trial to trial, the classification module does not possess enough information to make a correct decision.

Example (*primary outcome name*):

The target concentration required and number of target increments were noted at each step, as well as the total amount of drug needed until the trachea was successfully intubated and the total duration of the procedure.

### Fragment level

#### 3. Control interventions not clearly defined

Generally, experimental and control interventions are listed in the same sentence and contrasted with English expressions such as "either... or". The last intervention in the sentence usually, but not always, refers to the control treatment. Occasionally authors do not make a clear distinction between the experimental and control treatments in the key intervention sentences. In such cases, the separation can only be made based on clinical background knowledge (e.g., control is usually the conventional treatment for a studied condition) or other parts of the publication.

Example (*name of experimental and control treatments*):

Patients were randomly assigned (in a 1:1:1:1 ratio with the use of a two-by-two factorial design) to receive **goserelin **(3.6 mg given subcutaneously every 28 days) **plus either tamoxifen **(20 mg per day given orally) **or anastrozole **(1 mg per day given orally), **with or without zoledronic acid **(initially 8 mg given intravenously every 4 weeks).

#### 4. Linking multiple answers between related elements

Often, clinical trials compare the effects of several interventions, each with its own set of descriptors, i.e. dosage, frequency, route, and duration. Also, primary and secondary outcomes may each be assessed at different time points. An automatic system should ideally link the corresponding values for these elements. This part of the project has not yet been implemented.

### Limitations

Both the design of the system and the design of its evaluation account for several limitations to the current work.

Rather than targeting all types of human studies, we concentrated on randomized controlled drug treatment trials and excluded crossover trials, cluster-randomized trials and sub-studies of trials. This restriction on study type also, naturally, limits the set of information elements to be extracted. Additional information elements become relevant when further types of studies are included. The HSDB project, of which the current study is a part, does aim to capture the details of all completed and ongoing human studies, including observational and interventional, single and parallel group, crossover, cohort, and other types of study designs. While our proposed system is a general framework, minor modifications and extensions may be beneficial for other types of studies (e.g., observational studies) and information elements (e.g., design type [39]).

As mentioned before, our task and document characteristics differ from other studies, making a direct comparison difficult. Lacking benchmark datasets, we resorted to our own evaluation around manual verification of the automatically extracted information. The expense of manual curation poses limits on what sample size is practically feasible. We believe that our testing sample size (1050 extraction tasks, derived from 50 test documents from 25 journals) was appropriate given the result consistency with the leave-one-out cross-validation on the training set (2772 extraction tasks derived from 132 training documents from 22 journals). Frequencies for some information elements - notably, *early stopping *and *funding number *- were small, and the system performance measurement on these may be less reliable than on other information elements. In our study we did not attempt to assess the performance of certain separate sub-components such as the section detection method, since requirements for sample size and data collection for such an evaluation would differ greatly from those for the main evaluation. For the same reason, we did not attempt to reliably measure usability factors and time savings for curators using ExaCT.

Finally, a number of potentially beneficial research directions have not been explored in the present work. These include rhetorical structure identification, part-of-speech and dependency parsing, and the use of specialized vocabularies. Further research and experimentation may reveal a value of these techniques for the task at hand.

### Future work

The primary source documents in the current work have been full-text journal articles reporting on completed trials. In the future, we plan to adapt the same approach to information extraction from other sources, primarily to protocol documents, but also to conference abstracts and trial registration entries. During the life span of a clinical trial, several documents describing its goals, design characteristics, realization, and results may be written to meet various planning and regulatory requirements. The format and content for each type of document vary significantly. Some are rigidly structured (e.g. registration entries) while others are more free-form. Extracting key trial characteristics from multiple document sources as early as possible in a trial's lifecycle, and again at subsequent critical lifecycle junctures such as journal publication, would allow for the most thorough capture of trial information for comprehensive computational support of the clinical trial knowledge management as envisioned in the HSDB Project.

## Conclusions

This paper presents a working system, ExaCT, that assists curators in extracting key trial characteristics from journal articles. The system is comprised of two main parts: IE engine and interactive user interface. The IE engine automatically identifies pieces of text in a journal publication that describe the trial's interventions, population, outcome measures, funding sources, and other characteristics (information elements). A uniform two-stage process, in which target sentence identification is followed by application of weak extraction rules, is applied to full-text articles. The top-scored sentences with target snippets highlighted for each of the 21 information elements are presented to a curator through the user interface. The curator assesses and corrects the information before it is stored in the database.

The evaluation conducted on 50 previously unseen RCT articles confirmed the applicability and efficacy of the system. In 94% of the test tasks (992/1050), the automatic IE engine was able to return a fully correct or partially correct solution. The user interface provided the curator a satisfying tool to review suggestions in the context of the source article. In 58 tasks (less than 6%), the curator discarded the system suggestion and chose to manually locate the correct answer. In 296 tasks (28%), the curator made changes to the suggestions; for many of these tasks the change was minor (n = 109). In over 66% of the tasks (n = 696), the solutions were fully correct and complete, and required only confirmation from the curator. These results indicate the system's potential for considerable savings in curation time and promise efficiency gains for systematic reviewers of the literature such as meta-analysts and guideline developers.

## Availability and Requirements

**Project name**: ExaCT

**Project home page**: http://exactdemo.iit.nrc.ca

**Operating systems**: web-based, platform-independent

**Programming languages**: PHP, JavaScript, Java

**Other requirements**: JavaScript-enabled browsers, e.g. Fire Fox 1.0 or higher, IE 5.5 or higher

**License**: a demo version available on the project home page illustrates the capabilities of the ExaCT system; to use parts of the code for another purpose under an academic license or to obtain a commercial license, contact the authors.

## Competing interests

The authors declare that they have no competing interests.

## Authors' contributions

SK participated in the study design and implementation and drafted the manuscript. BdB devised the study and participated in its implementation. SC participated in the design of the study, collected and prepared the data, and performed the system evaluation. JM initiated the study, participated in its design and coordination, and was involved in the system implementation. IS initiated the study, participated in its design and coordination, and was involved in the system evaluation. All authors read and approved the final manuscript.

## Authors' information

SK and BdB are Research Officers at the Institute for Information Technology (IIT), National Research Council of Canada (NRC). SC is a Programmer/Analyst at the Division of General Internal Medicine, University of California San Francisco (UCSF). JM is a Senior Research Officer at IIT NRC. IS is an Associate Professor of Medicine at the Division of General Internal Medicine, UCSF and the Director of the Center for Clinical and Translational Informatics, UCSF.

## Pre-publication history

The pre-publication history for this paper can be accessed here:

http://www.biomedcentral.com/1472-6947/10/56/prepub

## Supplementary Material

Additional file 1 **Demonstration of the features of the interactive user interface in ExaCT (ExaCTDemo.mp4)**. A 5-minute video in MPEG-4 (MP4) format demonstrating the main features of ExaCT's user interface. The file can be viewed with any modern media player capable of playing MP4 files (e.g. QuickTime Player, RealPlayer). Dimensions: 640 × 480. Click here for file
